# Translation, Cross-Cultural Adaptation, and Validation of the Croatian Version of the SARC-F Questionnaire for Assessing Sarcopenia in Older Adults

**DOI:** 10.3390/healthcare14020151

**Published:** 2026-01-07

**Authors:** Edina Pulić, Ivna Kocijan, Mirjana Telebuh, Ivan Jurak, Tatjana Njegovan Zvonarević, Lana Feher Turković, Vlatko Brezac, Želimir Bertić, Miljenko Franić, Klara Turković, Ana Mojsović Ćuić

**Affiliations:** 1 Department of Occupational Therapy, University of Applied Health Sciences, Mlinarska Street 38, 10000 Zagreb, Croatia; edina.pulic@zvu.hr (E.P.); tatjana.njegovan-zvonarevic@zvu.hr (T.N.Z.); 2 Department of Anatomy and Physiology, University of Applied Health Sciences, Mlinarska Street 38, 10000 Zagreb, Croatia; 3 Department of Physiotherapy, University of Applied Health Sciences, Mlinarska Street 38, 10000 Zagreb, Croatia; mirjana.telebuh@zvu.hr (M.T.); ivan.jurak@zvu.hr (I.J.); 4 Department of Physiotherapy, Alma Mater Europaea University, Slovenska Street 17, 2000 Maribor, Slovenia; 5 Department of Chemistry, Biochemistry and Clinical Chemistry, University of Applied Health Sciences, Mlinarska Street 38, 10000 Zagreb, Croatia; lana.feher-turkovic@zvu.hr; 6 Department of Social Gerontology, Alma Mater Europaea University, Slovenska Street 17, 2000 Maribor, Slovenia; brezac.vlatko@gmail.com; 7 Institute of Public Health of Bjelovar-Bilogora County, Division of Public Health, Matice Hrvatske 15, 43000 Bjelovar, Croatia; bertic.z@gmail.com; 8 Department of Nursing, Faculty of Health Studies, University of Rijeka, Viktora Cara Emina 5, 51000 Rijeka, Croatia; 9 Department of Clinical Medicine, University of Applied Health Sciences, Mlinarska Street 38, 10000 Zagreb, Croatia; mfranic@kbd.hr; 10 Department of Orthopedics and Traumatology, Dubrava University Hospital, Avenija Gojka Šuška 6, 10000 Zagreb, Croatia; 11 Department of Orthopaedics, School of Medicine, University of Zagreb, Šalata 3, 10000 Zagreb, Croatia; 12 Institute for Integrative Medicine, Faculty of Dental Medicine and Health, J.J. Strossmayer University of Osijek, Crkvena 21, 31000 Osijek, Croatia; 13 Health Care Institution ‘Medici’, 10000 Zagreb, Croatia; kt.turkovic@gmail.com; 14 Department of Biology and Physics, University of Applied Health Sciences, Mlinarska Street 38, 10000 Zagreb, Croatia

**Keywords:** SARC-F, sarcopenia, validation, reliability, muscle strength, aging, Croatia

## Abstract

**Background/Objectives:** Sarcopenia is a growing public health challenge in older adults, being associated with functional decline, frailty, and increased mortality. The SARC-F questionnaire is a widely recommended screening tool for sarcopenia; however, no validated Croatian version has been available so far. This study aimed to translate, culturally adapt, and validate the Croatian version of the SARC-F questionnaire for older adults. **Methods:** In a cross-sectional design, 153 participants aged ≥ 65 years from Zagreb and Bjelovar were enrolled between March and September 2025. Psychometric evaluation included internal consistency (Cronbach’s α), test–retest reliability (intraclass correlation coefficient, ICC), item–total correlations, and split-half reliability. Convergent validity was assessed via correlations with handgrip strength (HGS), Short Physical Performance Battery (SPPB), and timed up-and-go (TUG) tests. Known groups and construct validity were also examined. **Results:** The Croatian SARC-F showed good internal consistency (Cronbach’s α = 0.76; 95% CI: 0.70–0.82), with item–total correlations ranging from 0.34 (falls) to 0.80 (stairs) and excellent test–retest reliability (ICC = 0.86). Strong correlations were found with SPPB (ρ = −0.50; *p* < 0.001), TUG (ρ = 0.50; *p* < 0.001), and handgrip strength (ρ = −0.42; *p* < 0.001), supporting convergent validity. An exploratory factor analysis indicated a unidimensional structure explaining 43% of the variance. **Conclusions:** The Croatian version of SARC-F is a reliable, valid, and clinically feasible tool for identifying older adults who are at risk of sarcopenia. The results support its use in national screening and cross-cultural research across Europe.

## 1. Introduction

Sarcopenia is a progressive, aging-related decline in skeletal muscle mass and function. It is prevalent in older adults, affecting mobility, functional ability, independence, and quality of life and increasing the risk of cardiac and respiratory disease, injuries, falls, fractures, and death. The presence of sarcopenia can be a fundamental contributor to physical frailty [1]. The primary diagnostic criterion for sarcopenia and a reliable predictor of adverse clinical outcomes is reduced muscle strength, as recognized by the European Working Group on Sarcopenia in Older People (EWGSOP2) in its 2018 revised and updated consensus [2].

International expert groups, including the EWGSOP, recommend the use of screening tools that are feasible in daily clinical practice and community-based settings. Among the available options, the SARC-F questionnaire has gained prominence due to its minimal equipment requirements and straightforward scoring [3]. Originally developed in 2013 in the United States by Malmstrom and Morley [4], it was designed as a simple, rapid screening tool for use in geriatric and sarcopenia research to identify individuals who are at risk of sarcopenia, particularly among community-dwelling older adults. Accordingly, the SARC-F questionnaire assesses five functional items associated with sarcopenia: muscle weakness, impaired mobility, difficulty rising from a chair, stair-climbing limitations, and falls. These items reflect core biomechanical and neuromuscular components of movement that deteriorate early on in sarcopenia. Rising from a chair and stair-climbing performance rely heavily on the action of lower-limb muscles, particularly concentric quadriceps strength, the rate of force development, efficient neuromuscular activation, proprioceptive feedback, postural control, and inter-limb coordination, all of which decline with aging and muscle weakness [5,6]. Likewise, gait-related impairments reflect deficits in integrated muscle strength and neuromuscular coordination, representing clinically meaningful manifestations of sarcopenia that can be efficiently captured by the SARC-F and supporting its use for early identification in aging populations [7]. As a result, SARC-F captures functionally meaningful impairments based on muscle physiology and movement biomechanics. Furthermore, the tool’s scoring system shows a close correlation with two important indicators of muscle function: handgrip strength and gait speed. Higher SARC-F scores are generally associated with reduced handgrip strength and slower gait speed, indicating functional decline linked to sarcopenia and helping to identify individuals who may need further assessment using more accurate techniques such as dual X-ray absorptiometry (DXA), computed tomography (CT), magnetic resonance imaging (MRI), and sonography [8,9,10,11,12]. Beyond these associations, empirical studies have demonstrated that SARC-F scores align with performance across validated functional tests used in geriatric and clinical research. Individuals with higher scores show compromised lower-extremity function and reduced overall physical capacity in objective assessments, such as the Short Physical Performance Battery (SPPB) [13,14]. SARC-F test scores are also associated with psychological variables: Poorer SARC-F scores are linked to worse self-reported quality of life, particularly in domains relating to physical functioning [15,16]. In addition, impaired mobility, fear of falling, and loss of independence are associated with a higher prevalence of depressive and anxiety symptoms in older adults with higher SARC-F scores [17,18].

The practical utility of the SARC-F questionnaire lies primarily in its simplicity, as it does not require any equipment. It allows the risk of sarcopenia to be assessed within a very short time based on five questions and serves as the first step in the EWGSOP diagnostic algorithm. Accordingly, clinicians can use it for the rapid assessment of older individuals who are at risk of sarcopenia [19,20]. It can be administered repeatedly over time, suggesting its potential utility in longitudinal monitoring of functional status during rehabilitation [21]. In addition, the test has high specificity, meaning that it effectively excludes sarcopenia in individuals without symptoms or in those who score ≤4 points on the questionnaire [4,20,21]. It has been shown to be effective in predicting adverse outcomes, such as an increased risk of hospitalization, disability, falls, and mortality [21,22]. This is clinically important, as it enables early identification of patients who may benefit from preventive interventions and targeted care, even when a full diagnosis of sarcopenia according to strict criteria has not yet been established [2]. Its relevance for patients and the general population lies in its accessibility and ease of use, as it allows individuals to self-assess their risk of sarcopenia. In the case of an unfavorable result, this may encourage individuals to consult a physician and initiate appropriate interventions, such as therapeutic exercise and adequate nutritional support [4,23]. The wider use and availability of the questionnaire may also help raise awareness of sarcopenia as an important public health issue, particularly in the geriatric population.

Population studies show significant variation in sarcopenia prevalence among older adults depending on diagnostic criteria, with pooled global estimates ranging from 10% to 27% in individuals aged ≥ 60 years [24]. However, at present, no publicly accessible national data or registry on sarcopenia prevalence is available in Croatia. Common lifestyle and clinical factors among older Croatian adults, including insufficient physical activity, nutritional inadequacy, overweight/obesity, and cardiometabolic disease, are well-established contributors to sarcopenia development and highly prevalent in aging populations in countries that are similar to Croatia [25]. Sarcopenia’s association with various adverse health outcomes and heightened healthcare utilization, including greater demand for hospital services, hospitalization, nursing home admission, and long-term care, highlights its growing public health and economic impacts across Europe [2]. This reinforces the need for a validated Croatian SARC-F tool to facilitate early identification and intervention within clinical and community settings. According to the Croatian Bureau of Statistics, the average age of the total population in Croatia is 44.5 years, placing the country among the oldest nations in Europe. In 2024, the proportion of persons aged 65 years and older across different counties in Croatia ranged from 20.8% to 28.5% [26]. As Croatia faces rapid demographic aging, similarly to many other European nations, the recognition and management of sarcopenia are becoming increasingly important public health challenges.

While the Croatian language is the official language of Croatia, it is also widely understood across the South Slavic region—including Bosnia and Herzegovina, Serbia, and Montenegro—and, to a lesser extent, in Slovenia, North Macedonia, and Bulgaria. To date, no formal translation or validation of the SARC-F questionnaire has been conducted for the Croatian-speaking population or for any other country in the broader South-Eastern European region. This lack of a standardized and culturally adapted tool limits both clinical screening and the cross-country comparability of sarcopenia research. Establishing a validated Croatian version of the SARC-F questionnaire [27] is therefore essential not only for national implementation but also for strengthening regional and European efforts toward harmonized sarcopenia assessment.

As observed in previous SARC-F validation studies across different languages and populations, the availability of locally validated tools is essential to ensure accurate measurement, reproducibility, and relevance to the target population. The SARC-F questionnaire has undergone cross-cultural translation and validation in numerous languages, including Polish, Spanish, Portuguese, Greek, Turkish, Italian, French, Arabic, and Brazilian Portuguese [15,28,29,30,31,32,33,34,35,36,37,38]. Across different language versions, validation studies show good reliability and high specificity for ruling out sarcopenia but generally low-to-moderate sensitivity. Overall, these studies support the use of SARC-F as a brief, reliable screening tool for sarcopenia, while also highlighting its limitations in terms of sensitivity. The consistency of findings across languages provides a strong empirical basis for further translation and validation studies. Accordingly, the present study aimed to translate, culturally adapt, and validate the Croatian version of the SARC-F questionnaire for older adults. Specifically, we sought to evaluate its reliability (internal consistency and test–retest reliability), convergent and known-groups validity, and structural validity. Based on previous validation studies in other languages [21,31,35,38], we hypothesized that the Croatian SARC-F would show a predominantly unidimensional structure, with a single factor underlying the five items.

## 2. Materials and Methods

### 2.1. Study Design and Population

This study was conducted in two regions, the city of Zagreb and the town of Bjelovar in Bjelovar-Bilogora County, over a seven-month period from March to September 2025. Participants were recruited from four locations: 60 residents from the “Retirement Home Centar” and “Retirement Home Park” (City of Zagreb County), 43 residents from the “Retirement Home Bjelovar” (Bjelovar-Bilogora County), and 28 community-dwelling older adults from the “Association of Retirees Bjelovar, Branch Office–Community Center ‘Dr. Ante Starčević’” (Bjelovar-Bilogora County). A total of 153 participants (32 men and 121 women) with a mean age of 81.4 years (SD = 7.69) were enrolled. The sample size was determined based on methodological recommendations for validation studies, which suggest a minimum of 5–10 participants per item to ensure adequate factor stability. Given the five items of the SARC-F, a minimum of 50 participants was required. Participants were recruited through direct invitation at the aforementioned retirement homes and community centers. A retirement home staff member announced the study to residents and invited interested residents to participate. Eligibility was determined using criterion-based sampling: eligible participants were aged 65 years or older, able to stand and walk independently (with or without assistive devices), and capable of understanding verbal instructions. All participants provided written informed consent prior to data collection. Individuals with acute illnesses or severe cardiovascular, respiratory, orthopedic, neurological, or psychiatric disorders that could interfere with testing were excluded. Bedridden participants and those with cognitive impairment were also excluded. Three individuals were removed from the sample due to not meeting the minimum age criterion (<65 years).

### 2.2. Translation and Adaptation Procedure

The translation and cross-cultural adaptation of the SARC-F questionnaire followed the World Health Organization (WHO) guidelines for the translation and adaptation of health instruments (WHODAS 2.0) [39]. Independent forward translations were produced by two bilingual translators, reconciled by an expert panel, and back-translated by a native English speaker who was fluent in Croatian. A pilot test among 10 older adults evaluated its clarity, comprehension, and cultural relevance. Subsequently, cognitive interviewing using a brief think-aloud procedure and targeted probing was conducted to identify unclear or culturally inappropriate wording. The expert panel—comprising a gerontologist, occupational therapist, physiotherapist, and orthopedic clinician—reviewed all feedback and applied predefined criteria for item revision, including (1) semantic equivalence, (2) conceptual accuracy, (3) linguistic simplicity and readability for older adults, and (4) cultural appropriateness. Based on these criteria, the panel reached a consensus on minor wording adjustments to ensure that the Croatian version preserved the intent of the original instrument while remaining understandable and culturally relevant to the target population.

### 2.3. Questionnaire and Data Collection

The SARC-F questionnaire consists of five items addressing symptoms related to sarcopenia [4]: strength (difficulty lifting or carrying a 4.5 kg weight), assistance in walking (difficulty walking across a room), rising from a chair (difficulty standing up from a chair or bed), climbing stairs (difficulty climbing 10 stairs), and falls (number of falls in the past year). Each component is scored from 0 to 2 (0 = no difficulty; 1 = some difficulty; 2 = severe difficulty or impossible to perform), except for falls (0 = no falls; 1 = 1–3 falls; 2 = ≥4 falls). The total score ranges from 0 to 10, with a score ≥ 4 indicating a risk of sarcopenia. Data were collected using an orally administered, interviewer-led questionnaire, allowing for immediate clarification of item wording when necessary, and included participant characteristics and the Croatian SARC-F [27].

### 2.4. Additional Measures

Additional measurements were taken, and performance tests were conducted concurrently with the SARC-F. Handgrip strength (HGS): The mean handgrip strength was calculated as the average of three consecutive measurements using the “Saehan DHD-1 Digital Hand Dynamometer” (serial no. 000731). Cut-off values for sarcopenia risk were <16 kg for women and <27 kg for men [40]. The reliability and validity of the instrument have previously been established [41]. Handgrip strength was measured on the dominant hand, as recommended in sarcopenia research protocols [40,41]. Participants were assessed in a seated position, with shoulders adducted, elbow flexed at 90°, forearm in a neutral position, and wrist in 0–30° extension. Three consecutive trials were performed, with a brief rest between attempts, and the mean value was used for analysis. If the dominant hand could not be tested due to contraindications, the non-dominant hand was evaluated using the same protocol.

Anthropometric measurements: Height (cm) was measured without shoes using a stadiometer. Body weight (kg) was measured to the nearest 0.1 kg, and height to the nearest 0.1 cm. Forearm and calf circumference were measured with an inelastic tape. Body mass index (BMI) was calculated as weight (kg) divided by height squared (m^2^). Anthropometric procedures followed the standardized WHO measurement guidelines for epidemiological surveys, as outlined in the WHO STEPS Surveillance Manual [42]. Height was measured with participants standing upright, with their heels together and their head positioned in the Frankfort plane. Weight was assessed in light clothing and without shoes. Circumference measures (forearm, calf) were taken at standardized anatomical landmarks with a non-elastic tape, ensuring horizontal alignment and minimal tissue compression. Short Physical Performance Battery (SPPB): The SPPB is a widely validated tool assessing lower-extremity function through balance, gait, and chair stand tests [43,44]. The SPPB includes three subtests: (1) balance (side-by-side, semi-tandem, tandem stance), (2) 4 m gait speed, and (3) five-times chair stand. Each subtest is scored as 0–4 using established performance cut-offs, producing a total score from 0 to 12, where higher scores indicate better lower-extremity function [43,45]. Timed up-and-go (TUG) test: The TUG test measures functional mobility, balance, and gait speed. Participants started seated in a chair, stood up, walked three meters, turned, and returned to the chair. Assistive devices were permitted if regularly used [46].

### 2.5. Statistical Analysis

All analyses were pre-specified to evaluate the psychometric properties of the Croatian version of the SARC-F questionnaire. Descriptive statistics were presented as means and standard deviations (SDs), medians with ranges, and frequencies with percentages, as appropriate. Normality was assessed visually and with the Shapiro–Wilk test; given the non-normal distribution of most variables, nonparametric tests were applied. Internal consistency was evaluated using Cronbach’s alpha, corrected item–total correlations, alpha-if-item-deleted, average inter-item correlation, and split-half reliability (Guttman λ coefficients). Test–retest reliability was assessed in a subsample (*n* = 31) using the intraclass correlation coefficient (ICC, two-way agreement, single measures) and visualized with Bland–Altman plots showing the mean bias and 95% limits of agreement. Convergent validity was examined with Spearman’s correlation coefficients (ρ) between the SARC-F total score and objective measures of physical performance and muscle strength, including SPPB (total and subtests), TUG, and handgrip strength. Known-groups validity was assessed by comparing SARC-F scores between men and women using the Mann–Whitney U test with rank-biserial effect sizes. Construct validity was explored through exploratory factor analysis (principal axis factoring, oblimin rotation), with the adequacy of the correlation matrix being verified via the Kaiser–Meyer–Olkin (KMO) measure and Bartlett’s test of sphericity under the a priori assumption of a predominantly unidimensional structure. Model fit was evaluated using standard indices (CFI, TLI, RMSEA, and SRMR). All statistical tests were two-tailed, with α = 0.05. Analyses were performed using R software (Vienna, Austria, version 4.3.1) and jamovi (Sydney, Australia, version 2.3.28). Missing data were below 2% for all items, and a complete case analysis was applied.

### 2.6. Ethical Considerations

Ethical approval for this study was obtained from the Ethics Committee of the University of Applied Health Sciences, Zagreb, Croatia (29 December 2023; CLASS: 602-03/23-18/933; REG. NO.: 251-379-10-23/02), the Ethics Committee of the City of Zagreb (11 March 2025; CLASS: 550-01/25-001/130; REG. NO.: 251-09-12-2/003-25-4), and the Administrative Board of the Bjelovar Pensioners’ Association (3 March 2025). All participants were fully informed about the study objectives and procedures, confidentiality was maintained throughout the research process, and written informed consent was obtained from each participant, in accordance with the approved study protocol and the principles of the Declaration of Helsinki.

## 3. Results

### 3.1. Descriptive Statistics

Table 1 summarizes the baseline demographic, anthropometric, and strength characteristics of the study sample (N = 153). Participants with a risk of sarcopenia (*n* = 81) were on average older (82.9 vs. 79.6 years) and had higher BMI values (28.7 vs. 26.7) than those without risk. The gender distribution differed, with women comprising 86.4% of the risk group versus 70.8% in the no-risk group. No major differences were observed in height, weight, or forearm circumference. Calf circumference was slightly smaller in the risk group (37.7 vs. 39.0 cm), while handgrip strength was markedly lower in participants who were at risk (mean 19.6 vs. 23.5 kg), where the prevalence of weak grip strength was more than twice as high (29.6% vs. 13.9%).

Table 2 presents descriptive parameters of the SARC-F, SPPB, and TUG tests’ performances. Participants who were at risk of sarcopenia had substantially higher SARC-F scores across all items, with a mean overall score of 5.91 compared with 1.36 in the no-risk group. Performance-based measures also differed: the risk group demonstrated poorer outcomes in the SPPB, with lower balance (1.84 vs. 2.68), gait (2.23 vs. 3.10), and chair stand scores (1.15 vs. 2.25), resulting in a lower total SPPB score (5.30 vs. 8.11). Similarly, TUG times were markedly prolonged in the risk group (19.6 vs. 11.9 s), indicating reduced mobility. These findings confirm consistent differences in both self-reported and performance-based measures between the groups.

### 3.2. Reliability

As shown in Table 3, the Croatian version of the SARC-F demonstrated acceptable internal consistency, with Cronbach’s α = 0.76 (95% CI: 0.70–0.82). Item–total correlations ranged from 0.34 (falls) to 0.80 (stairs), with the falls item contributing the least to the overall scale. Removing item 5 (falls) would slightly increase α to 0.79, but the item was retained for clinical relevance. Split-half reliability estimates were consistent with these findings (λ4 = 0.80; average = 0.72).

### 3.3. Convergent Validity

As shown in Table 4, the SARC-F total score demonstrated significant correlations with objective performance measures. Higher SARC-F scores were moderately associated with poorer physical performance, reflected by lower SPPB scores (ρ = −0.50; *p* < 0.001) and longer TUG times (ρ = 0.50; *p* < 0.001). In addition, SARC-F correlated negatively with handgrip strength (ρ = −0.42; *p* < 0.001). The associations among the performance tests were also strong, with SPPB being strongly inversely related to TUG (ρ = −0.68; *p* < 0.001) and moderately positively related to grip strength (ρ = 0.43; *p* < 0.001). These findings support the convergent validity of the Croatian SARC-F, confirming its alignment with established measures of muscle strength and physical function.

### 3.4. Test–Retest Reliability

**Figure 1:**
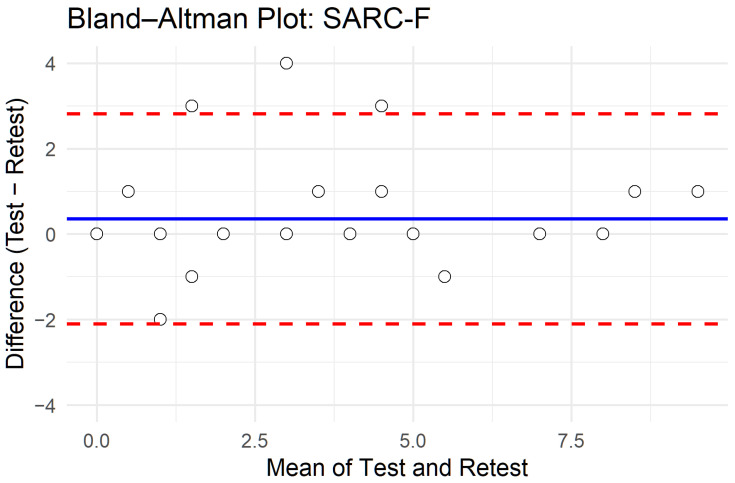
Bland–Altman plot of test–retest agreement for SARC-F total score.

Table 5 presents the intraclass correlation coefficients (ICCs, two-way agreement, single measures) of individual items and the total score. ICCs ranged from 0.671 (falls) to 0.889 (strength), with the total score showing excellent reliability (ICC = 0.862; 95% CI: 0.730–0.931; *p* < 0.001). Figure 1 shows the corresponding Bland–Altman plot for the total score, with a negligible mean bias and 95% limits of agreement from −2 to +3 points. The combined results indicate good stability of the SARC-F across repeated administrations, with only minor variability at the item level, particularly for the falls item.

### 3.5. Known-Groups Validity

As shown in Table 6, women scored significantly higher than men on the SARC-F1 items (mean of 1.20 vs. 0.50) and the SARC-F4 item (mean of 1.13 vs. 0.53). The total SARC-F score was also significantly higher in women (mean of 4.18 vs. 2.22). No significant gender differences were observed for the SARC-F2, SARC-F3, or SARC-F5 items, where mean values were modestly higher in women but did not reach statistical significance.

### 3.6. Construct Validity

Factor analysis was also applied to the SARC-F. The sampling adequacy was acceptable (overall KMO = 0.74; item MSAs: 0.69–0.91), and Bartlett’s test of sphericity was highly significant (χ^2^(10) = 217.66; *p* < 0.001), confirming sufficient inter-item correlations. Parallel analysis suggested a two-factor structure, but extraction of a single factor was examined for consistency with the intended screening use. The one-factor model explained 43% of the variance, with the strongest standardized loadings for climbing stairs (0.84) and rising from a chair (0.71), while the falls item showed a weaker loading (0.34). Model fit indices were varied (CFI = 0.92; TLI = 0.83; RMSEA = 0.15), indicating mixed structural validity (strong CFI but lower TLI and elevated RMSEA); this pattern is commonly observed in very short scales, where global fit statistics tend to be overly sensitive to item count. Importantly, the factor loadings showed that the core functional items (stairs and rising from a chair) contributed strongly to the latent construct, supporting the intended unidimensional screening use. Despite the statistical limitations that are inherent to five-item models, the overall factor structure remains consistent with the practical purpose of SARC-F as a brief, clinically oriented screening tool for sarcopenia risk.

## 4. Discussion

The present study introduces and validates the first Croatian version of the SARC-F questionnaire, representing the first psychometric evaluation of this tool among the South Slavic languages. This constitutes an important advancement for the early detection and management of sarcopenia in Croatia and the wider South Slavic region, where standardized screening instruments have been lacking. The Croatian SARC-F demonstrated satisfactory internal consistency (Cronbach’s α = 0.76) and excellent test–retest reliability (ICC = 0.86), which is in line with the psychometric properties reported in other European adaptations. Comparable levels of internal consistency were documented in the Spanish version, with α = 0.779 [35], and the Polish version, with α = 0.784 [38], while for the German adaptation, α values ≥ 0.65–0.70 were considered acceptable [32]. Similarly, the observed test–retest reliability is consistent with that reported for the Greek version, with ICC = 0.93 [36]; the French version, with ICC = 0.86 [30]; and the German version, with ICC = 0.899 [32], supporting the robustness and cross-cultural stability of the instrument. The relatively weaker contribution of the “falls” item to the overall internal consistency reflects previous findings, including a Turkish proxy-administration study by Özkök et al. [47], in which this item showed the lowest item–total correlation (r = 0.443). This suggests that “falls” may encompass a broader construct associated with frailty, beyond muscle function itself. Although the SARC-F is designed and used as a brief unidimensional screening tool, we conducted an exploratory factor analysis, primarily for completeness, to examine whether the items demonstrate sufficient common variance to justify the use of a total score. The results suggest that the SARC-F items share sufficient common variance to be treated as a single underlying construct, in line with the tool’s established role as a brief screening instrument. These findings confirm the robustness and reproducibility of the Croatian version. When interpreting the internal consistency results, it is important to acknowledge that the relatively weaker performance of the “falls” item may partly reflect the characteristics of our sample. If the prevalence of falls was lower than expected or clustered within a narrow range, the limited variance could reduce the item–total correlation, and the psychometric contribution may vary depending on the functional status and risk profile of the studied population. The correlation between SARC-F and handgrip strength in our sample was somewhat lower than that observed in other validation studies. Several factors may explain this: First, the participants in our cohort were notably older (mean age of 81.4 years) and demonstrated generally low physical functioning, which may reduce the variability in muscle strength and attenuate correlation coefficients. Second, age-related multimorbidity—which is highly prevalent in institutionalized and community-dwelling older adults—may influence self-reported functional limitations independently of handgrip strength, as noted in previous research on strength decline and sex-specific aging trajectories [48,49]. Finally, potential measurement constraints, such as reduced ability to exert maximal voluntary effort or discomfort during testing, could introduce measurement error. These factors should be considered when interpreting the strength of associations in this population. The moderate-to-strong correlations between SARC-F, SPPB, TUG, and handgrip strength support its convergent validity and reinforce its role as a simple, cost-effective, and clinically meaningful screening measure for functional decline in older adults.

The practical utility of SARC-F as an effective case-finding tool for sarcopenia in older adults was recently confirmed in a related study in Croatia conducted by our group, testing its predictive value in differentiating high-risk individuals across nursing home, outpatient, and community settings [50]. The findings showed that age was the dominant risk factor for sarcopenia, particularly among institutionalized older adults, which supports the current study’s conclusions and highlights the importance of early screening and prevention strategies in this population.

Given the high degree of mutual intelligibility among South Slavic languages—including Bosnian, Serbian, and Montenegrin—the Croatian version of SARC-F holds potential for wider regional application. Its linguistic and cultural accessibility enables its use in neighboring countries with minimal modification, enhancing the comparability of sarcopenia research and facilitating cross-border collaboration. To date, no validated SARC-F versions have been published for other South Slavic countries, making this adaptation a crucial contribution to regional harmonization efforts. This broader applicability supports the integration of standardized sarcopenia assessment tools across Europe, in accordance with recommendations from the EWGSOP2 group [2]. By adhering to the WHO guidelines for translation and cross-cultural adaptation of health instruments, this study ensured conceptual, linguistic, and metric equivalence with the original English version, strengthening both its clinical relevance and international comparability.

The significant correlations between SARC-F and objective measures of physical performance (SPPB and TUG) are in line with previous reports linking self-reported functional limitation to reduced muscle strength and mobility [44,45]. Gender-related differences in SARC-F scores should be interpreted with caution, as they may partly reflect sample characteristics such as age distribution and functional status. Nonetheless, the direction of these differences is consistent with epidemiological patterns showing that older women typically exhibit lower muscle strength [48,49] and greater functional limitations than men. These patterns support known-groups validity, although they do not imply that gender alone accounts for the observed differences.

From a clinical perspective, the Croatian SARC-F provides a practical, low-cost, and feasible screening option for community-dwelling older adults. Its rapid administration and ease of interpretation make it suitable for primary care and geriatric practice, where time and resources are limited. In accordance with the EWGSOP2 framework, which prioritizes muscle strength and physical performance as key diagnostic criteria, SARC-F can serve as a first-line screening instrument to identify individuals who require more detailed assessment through methods such as handgrip dynamometry or SPPB testing. Beyond its national relevance, this validation supports the establishment of standardized approaches to sarcopenia assessment in South-Eastern Europe and promotes early intervention strategies aimed at reducing frailty and disability in aging populations.

### Limitations

A key limitation is the absence of bioelectrical impedance analysis (BIA) or other imaging-based techniques for the direct assessment of muscle mass, which may have reduced the diagnostic precision of our study. This methodological decision was, however, consistent with the revised EWGSOP2 (2018) criteria, which highlight muscle strength and physical performance as the primary diagnostic components, while muscle mass serves as confirmatory evidence [2].

Another limitation is that participants were recruited from two urban regions in Croatia (Zagreb and Bjelovar), which may limit the generalizability of our findings to rural or institutionalized populations. Nevertheless, by including both community-dwelling and nursing home residents, the study encompassed a broad functional spectrum of older adults.

An additional limitation is that the prevalence of falls in our sample may have been lower than expected, resulting in limited variability of the falls item. This restricted variance may have attenuated the item–total correlation and partially influenced the psychometric performance.

Furthermore, according to a recent systematic review, the SARC-F demonstrates high specificity but low sensitivity across studies, raising concerns about its ability to detect individuals with early or milder manifestations of sarcopenia [51]. Although the SARC-F demonstrates good reliability and is easy to apply in clinical practice, its lower sensitivity may lead to under-recognition of early cases. This should be considered in future research and when considering the use of modified tools such as SARC-F EBM or SARC-F CalF [29,37,52].

Finally, the Bland–Altman analysis showed relatively wide limits of agreement for the SARC-F total score (approximately −2 to +3 points). Although the mean bias was negligible, this degree of variation implies that individuals whose baseline scores lie close to the screening threshold of 4 points may shift between “at risk” and “not at risk” classifications upon retesting. Such fluctuations are common in brief self-reported screening tools, especially when measuring symptoms or functional difficulties that can vary from day to day. Nevertheless, the overall test–retest ICC was excellent, indicating good stability at the group level; however, clinicians should exercise caution when interpreting small changes in SARC-F scores over time in individual patients.

## 5. Conclusions

This study represents the first translation, cultural adaptation, and validation of the SARC-F questionnaire in the Croatian language, marking the first psychometric evaluation in Croatia and within the South Slavic region. The Croatian SARC-F demonstrated good internal consistency, excellent test–retest reliability, and strong convergent validity with objective measures of muscle strength and physical performance. These findings confirm its reliability and suitability as a rapid, low-cost screening tool for identifying older adults who are at risk of sarcopenia in both clinical and community settings. By providing a culturally adapted and psychometrically robust instrument, this study contributes to the harmonization of sarcopenia assessment across Europe and establishes a foundation for future cross-cultural research. Implementation of the Croatian SARC-F may facilitate earlier detection, targeted interventions, and improved care for aging populations, aligning with the goals of EWGSOP2 to promote healthy and active aging.

## Figures and Tables

**Table 1:** Baseline demographic, anthropometric, and strength characteristics of the study sample by SARC-F risk status.

	Risk: No	Risk: Yes	Overall
	(N = 72)	(N = 81)	(N = 153)
Gender			
Male	21 (29.2%)	11 (13.6%)	32 (20.9%)
Female	51 (70.8%)	70 (86.4%)	121 (79.1%)
Age			
Mean (SD)	79.6 (8.28)	82.9 (6.81)	81.4 (7.69)
Median [Min, Max]	79.5 [65.0, 96.0]	84.0 [65.0, 97.0]	82.0 [65.0, 97.0]
Height			
Mean (SD)	166 (8.64)	164 (7.41)	165 (8.06)
Median [Min, Max]	167 [148.0, 185.0]	163 [150.0, 183.0]	164 [148.0, 185.0]
Weight			
Mean (SD)	74.0 (12.8)	77.0 (13.0)	75.6 (13.0)
Median [Min, Max]	74.0 [48.0, 105.0]	75.0 [50.0, 115.0]	75.0 [48.0, 115.0]
BMI			
Mean (SD)	26.7 (3.70)	28.7 (5.11)	27.8 (4.62)
Median [Min, Max]	26.3 [18.2, 34.6]	28.0 [19.3, 46.3]	27.3 [18.2, 46.3]
Forearm			
Mean (SD)	25.9 (3.32)	25.4 (3.13)	25.6 (3.21)
Median [Min, Max]	25.8 [20.0, 37.6]	25.0 [19.8, 33.0]	25.0 [19.8, 37.6]
Calf			
Mean (SD)	39.0 (5.0)	37.7 (5.82)	38.3 (5.47)
Median [Min, Max]	38.7 [28.0, 55.0]	37.0 [27.0, 59.0]	38.0 [27.0, 59.0]
Hand Grip Strength (HGS)			
Mean (SD)	23.5 (6.52)	19.6 (6.83)	21.4 (6.95)
Median [Min, Max]	23.2 [10.0, 41.1]	19.3 [3.20, 45.2]	21.1 [3.20, 45.2]
HGS—categorized			
Normal HGS	62 (86.1%)	57 (70.4%)	119 (77.8%)
Weak HGS	10 (13.9%)	24 (29.6%)	34 (22.2%)

**Table 2:** Descriptive parameters of SARC-F, SPPB, and TUG tests.

	Risk: No	Risk: Yes	Overall
	(N = 72)	(N = 81)	(N = 153)
SARC-F1 (Strength)			
Mean (SD)	0.5 (0.7)	1.5 (0.7)	1.1 (0.9)
Median [Min, Max]	0 [0, 2.0]	2.0 [0, 2.0]	1.0 [0, 2.0]
SARC-F2 (Assistance in walking)			
Mean (SD)	0.1 (0.4)	1.1 (0.7)	0.6 (0.8)
Median [Min, Max]	0 [0, 2.0]	1.0 [0, 2.0]	0.0 [0, 2.0]
SARC-F3 (Rising from chair)			
Mean (SD)	0.1 (0.3)	1.0 (0.7)	0.6 (0.7)
Median [Min, Max]	0 [0, 1.0]	1.0 [0, 2.0]	0 [0, 2.0]
SARC-F4 (Climbing stairs)			
Mean (SD)	0.3 (0.5)	1.5 (0.6)	1.0 (0.8)
Median [Min, Max]	0 [0, 2.0]	2.0 [0, 2.0]	1.0 [0, 2.0]
SARC-F5 (Falls)			
Mean (SD)	0.3 (0.5)	0.7 (0.7)	0.5 (0.7)
Median [Min, Max]	0 [0, 2.0]	1.0 [0, 2.0]	0 [0, 2.0]
SARC-F Overall score			
Mean (SD)	1.4 (1.2)	5.9 (1.8)	3.8 (2.8)
Median [Min, Max]	1.0 [0, 3.0]	6.0 [1.0, 10.0]	4.0 [0, 10.0]
SPPB: Balance test			
Mean (SD)	2.7 (1.5)	1.8 (1.5)	2.2 (1.6)
Median [Min, Max]	3.5 [0, 4.0]	2.0 [0, 4.0]	2.0 [0, 4.0]
SPPB: Gait test			
Mean (SD)	3.1 (0.9)	2.2 (1.0)	2.6 (1.1)
Median [Min, Max]	3.0 [1.0, 4.0]	2.0 [1.0, 4.0]	3.0 [1.0, 4.0]
SPPB: Chair test			
Mean (SD)	2.3 (1.2)	1.2 (1.3)	1.7 (1.4)
Median [Min, Max]	2.0 [0, 4.0]	1.0 [0, 4.0]	1.0 [0, 4.0]
SPPB			
Mean (SD)	8.1 (3.0)	5.3 (3.1)	6.6 (3.3)
Median [Min, Max]	8.5 [1.0, 12.0]	5.0 [1.0, 12.0]	6.0 [1.0, 12.0]
TUG			
Mean (SD)	11.9 (5.6)	19.6 (10.2)	16.0 (9.2)
Median [Min, Max]	11.0 [5.6, 45.3]	16.6 [8.2, 52.0]	12.7 [5.6, 52.0]

**Table 3:** Internal consistency: Cronbach’s alpha and split-half reliability.

Metric	Value
Cronbach’s α (raw/standardized)	0.76/0.76
Cronbach’s α if item dropped	0.74/0.70/0.70/0.65/0.79
95% CI for α (Feldt/Duhachek)	0.70–0.82/0.71–0.82
Average inter-item correlation (median)	0.39 (0.33)
Guttman (λ4/λ6/λ3/λ2/β)	0.80/0.76/0.76/0.77/0.62
Average split-half reliability	0.72
Item–total correlations	0.58/0.69/0.69/0.80/0.34

**Table 4:** Spearman correlations between SARC-F, SPPB, TUG, and HGS (df = 151).

Variable	SARC-F	SPPB	TUG	HGS
SARC-F	—	−0.496 (*p* < 0.001)	0.504 (*p* < 0.001)	−0.424 (*p* < 0.001)
SPPB		—	−0.684 (*p* < 0.001)	0.434 (*p* < 0.001)
TUG			—	−0.307 (*p* < 0.001)
HGS				—

**Table 5:** Test–retest reliability: intraclass correlation coefficients (ICCs, agreement, two-way model, single measures, *n* = 31).

SARC-F Item/Total	ICC (A,1)	95% CI	F (df1, df2)	*p*-Value
SARC-F1 (Strength)	0.889	0.782–0.945	F (30, 29.5) = 17.7	<0.001
SARC-F2 (Assistance in walking)	0.791	0.612–0.893	F (30, 30) = 8.9	<0.001
SARC-F3 (Rising from chair)	0.861	0.732–0.930	F (30, 30.3) = 13.1	<0.001
SARC-F4 (Climbing stairs)	0.844	0.702–0.922	F (30, 30.3) = 11.6	<0.001
SARC-F5 (Falls)	0.671	0.418–0.827	F (30, 30.1) = 4.96	<0.001
SARC-F Total Score	0.862	0.730–0.931	F (30, 28) = 14.3	<0.001

**Table 6:** Mann–Whitney U tests for gender differences in SARC-F items and total score.

Variable	U	*p*-Value	Rank Biserial r
SARC-F1 (Strength)	1108	<0.001	0.428
SARC-F2 (Assistance in walking)	1745	0.345	0.099
SARC-F3 (Rising from chair)	1615	0.106	0.166
SARC-F4 (Climbing stairs)	1171	<0.001	0.395
SARC-F5 (Falls)	1650	0.145	0.148
SARC-F Total Score	1150	<0.001	0.406

## Data Availability

The data presented in this study are available on request from the corresponding author. The data are not publicly available due to privacy restrictions.

## Abbreviations

The following abbreviations are used in this manuscript:

BIA	Bioelectrical impedance analysis
BMI	Body mass index
CFI	Comparative fit index
CT	Computed tomography
DXA	Dual X-ray absorptiometry
EWGSOP	European Working Group on Sarcopenia in Older People
HGS	Handgrip strength
ICC	Intraclass correlation coefficient
KMO	Kaiser–Meyer–Olkin measure
MRI	Magnetic resonance imaging
RMSEA	Root mean square error of approximation
SARC-F	Strength, Assistance in walking, Rise from a chair, Climb stairs, and Falls
SDs	Standard deviations
SPPB	Short Physical Performance Battery
SRMR	Standardized root mean square residual
TLI	Tucker–Lewis index
TUG	Timed up-and-go test
WHO	World Health Organization
